# CRISPR and Fanzor in sickle cell disease: current progress and future prospects

**DOI:** 10.3389/fgeed.2026.1774014

**Published:** 2026-05-26

**Authors:** Aisha Yousef Alhumoudi, Aminah Ghazi Alotaibi, Nada Fahad Alosaimi, Abdulrahman Alshalani, Saad M. Alqahtani, Sarah M. Alsaab, Basem Jahz Almutiri, Mohammed M. H. Albariqi

**Affiliations:** 1 Applied Genomics Technologies Institute, Health Sector, King Abdulaziz City for Science and Technology, Riyadh, Saudi Arabia; 2 Department of Biotechnology Evaluation and Development National Livestock and Fisheries Development Program, Riyadh, Saudi Arabia; 3 Department of Clinical Laboratory Sciences, College of Applied Medical Sciences, King Saud University, Riyadh, Saudi Arabia; 4 Digital Health Institute, Health Sector, King Abdulaziz City for Science and Technology, Riyadh, Saudi Arabia

**Keywords:** sickle cell disease, CRISPR, CRISPR-Cas9, Fanzor, genome editing, gene therapy

## Abstract

Advancements in genome editing have established a new frontier for the treatment of various genetic diseases, including sickle cell disease (SCD). SCD, the most prevalent monogenic blood disorder, causes severe pain, organ damage, and reduced life expectancy. The recent clinical approval of clustered regularly interspaced short palindromic repeats (CRISPR)-Cas9-based gene therapies for severe sickle cell anemia marks a significant milestone in treating genetic diseases. Despite these breakthroughs, limitations in CRISPR technology persist, requiring further innovation. Alternative approaches, such as the Fanzor (Fz) system, are being developed to complement CRISPR’s capabilities. Unlike CRISPR, which is typically encoded within prokaryotic organisms, Fz is encoded in the eukaryotic genome, offering a universal RNA-guided mechanism applicable across all life kingdoms. Fz’s eukaryotic origin may facilitate more efficient delivery across diverse cell types and tissues, enhancing its therapeutic potential. Here, we will review the current successes and limitations of the CRISPR technology in editing mutation associated with SCD. Additionally, we will explore the potential role of Fz as a genome-editing tool for SCD, a field where its application has not yet been studied.

## 1 Introduction

**FIGURE 1:**
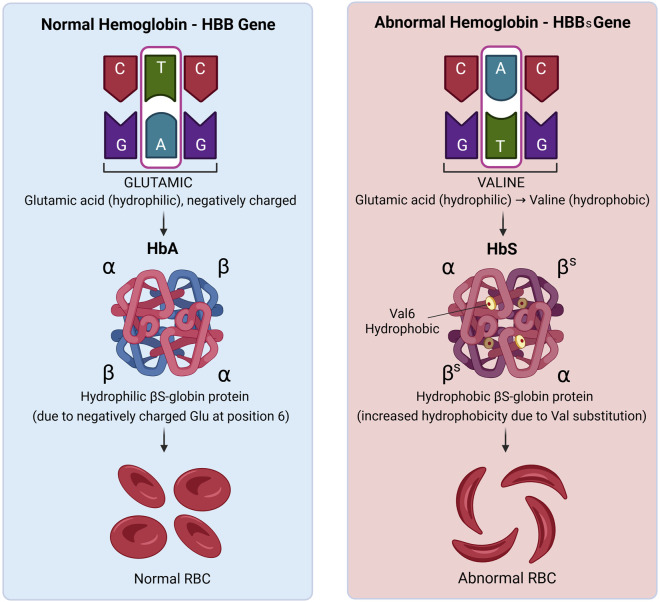
Genetic and molecular mechanisms underlying Sickle cell disease (SCD). SCD is caused by a single-point mutation in the β-globin gene (HBB). This mutation involves a nucleotide substitution from adenine (A) to thymine (T), which changes the codon from GAG to GTG, resulting in the replacement of glutamic acid with valine at the sixth position of the beta-globin chain. This amino acid change leads to the production of abnormal hemoglobin S (HbS) instead of normal hemoglobin A (HbA), which contributes to the characteristic sickling of red blood cells in individuals with SCD.

Sickle cell disease (SCD) is a genetic disorder caused by a single point mutation in the sixth codon of the HBB gene (beta-globin gene), resulting in the production of an abnormal hemoglobin protein, hemoglobin S (HbS) (Abraham and Tisdale, 2021). Hemoglobin S transforms flexible red blood cells into rigid, sickle-shaped cells (Figure 1) (Piel et al., 2017). These sickle cells can block blood flow, and result in pain and organ damage (Rees et al., 2010).

SCD affects around 515,000 newborns worldwide each year, with over 75% of cases occurring in Africa (Thomson et al., 2023). Without proper medical care, nearly 30% of untreated African children with SCD die before the age of five (Thomson et al., 2023). Although supportive care, hydroxyurea therapy, and blood transfusions have improved outcomes, these approaches remain largely palliative and do not address the underlying genetic defect (Riley et al., 2024; Guidelines for Red Blood Cell, 1997). Allogeneic hematopoietic stem cell transplantation (HSCT) offers a potential cure but is limited by donor availability, transplant-related risks, and graft-versus-host disease (Bhalla et al., 2023). These limitations underscore the urgent need for curative, gene-based therapeutic strategies.

Genome-editing technologies have emerged as a transformative approach for addressing monogenic disorders such as SCD by enabling precise modification of disease-causing genetic variants (Randhawa and Sengar, 2021; Commissioner, 2025). Since the advent of genetic engineering in the 1970s, the ability to manipulate DNA and RNA has revolutionized biomedical research and therapeutic development (Hsu et al., 2014). Modern genome-editing tools aim to achieve highly efficient, site-specific genetic modification with minimal off-target effects, particularly in clinically relevant cell types such as hematopoietic stem cells (Commissioner, 2025).

The rapid development of genome-editing techniques has been largely driven by the use of engineered or bacterial nucleases. A transformative breakthrough came with the discovery of the clustered regularly interspaced short palindromic repeats (CRISPR) system and its associated proteins (Cas) (Ishino et al., 1987). Over the past decade, advances in the CRISPR-Cas system have significantly expanded the potential of genome editing. This system has been widely adopted across agriculture, biotechnology, and medicine as a powerful genome-editing tool (Doudna and Charpentier, 2014). Notably, CRISPR-based genome editing has recently received regulatory approval for the treatment of SCD and transfusion-dependent β-thalassemia, marking a milestone in precision medicine (Parums, 2024).

Targeted genome editing relies on programmable nucleases that introduce site-specific DNA lesions, activating endogenous DNA repair pathways such as non-homologous end joining (NHEJ) and homology-directed repair (HDR) (Ray and Raghavan, 2020). In the context of SCD, genome-editing strategies have focused on correcting the pathogenic HBB mutation, disrupting regulatory elements to induce fetal hemoglobin expression, or modifying globin gene regulation to ameliorate disease severity. Increasing emphasis has been placed on improving editing precision, minimizing genotoxicity, and enhancing scalability for clinical translation (Ray and Raghavan, 2020).

Recent discoveries have further expanded the genome-editing toolbox. In 2021, the OMEGA (Obligate Mobile Element-guided Activity) system, a new class of prokaryotic RNA-guided systems was discovered in bacteria, marking another milestone in the field (Altae-Tran et al., 2021). Building on this momentum, the Fanzor (Fz) system was identified in 2023—as a family of eukaryotic CRISPR-Cas-like endonucleases (Saito et al., 2023). Unlike bacterial CRISPR-Cas systems, Fz proteins are naturally present in eukaryotes and are considerably smaller in size (approximately 400–700 amino acids), offering potential advantages for therapeutic delivery and intracellular stability (Saito et al., 2023).

**FIGURE 2:**
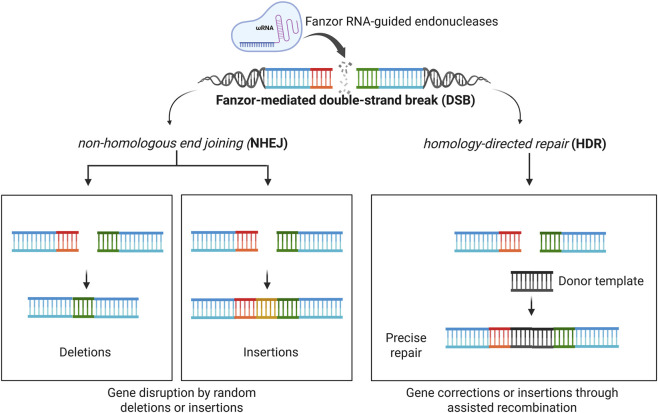
DNA repair mechanisms following Fanzor-induced double-strand breaks. Double-strand breaks (DSBs) generated by the Fanzor endonuclease are primarily repaired through two major cellular pathways: non-homologous end joining (NHEJ) and homology-directed repair (HDR). In NHEJ, the broken DNA ends are processed and rejoined without a homologous template, often using short microhomologous sequences; this can introduce insertions or deletions that disrupt the reading frame and inactivate the target gene. In contrast, HDR uses a donor DNA template with homology to the sequences flanking the break, enabling precise repair and allowing Fanzor-mediated genome editing to introduce defined mutations or insert new genetic material at the DSB site (see text for references).

Mechanistically, Fz generate double-stranded DNA breaks (DSBs) or staggered cuts at defined genomic loci, triggering endogenous DNA repair processes in mammalian cells (Ray and Raghavan, 2020). While classical repair pathways such as non-homologous end joining (NHEJ) and homology-directed repair (HDR) remain central to editing outcomes, contemporary strategies increasingly focus on controlling repair pathway choice, enhancing editing efficiency, and minimizing genotoxicity. In therapeutic applications, including sickle cell disease, genome editing is commonly leveraged to induce targeted gene disruption, precise sequence correction, or regulatory element modification, with growing interest in approaches that reduce reliance on HDR and improve safety and scalability for clinical translation (Figure 2) (Ray and Raghavan, 2020).

The compact size of Fz makes it particularly attractive for packaging into commonly used viral delivery systems such as adeno-associated viruses (AAVs), which is a critical consideration for efficient editing of bone marrow-derived hematopoietic stem cells in SCD (Tasan et al., 2016). In contrast, widely used CRISPR-associated nucleases such as Cas9 typically exceed 1300 amino acids, posing challenges for delivery efficiency and vector design (Sebastiano et al., 2011). Moreover, Fz’s natural presence in eukaryotic cells suggests it may interact more harmoniously with the cell’s intrinsic DNA repair pathways—homologous recombination (HR) and non-homologous end joining (NHEJ)—compared to bacterial-origin systems such as CRISPR-Cas9. This compatibility could improve both the precision and efficiency of β-globin gene correction in SCD.

In this review, we focus on recent advances in genome-editing approaches for sickle cell disease, with particular emphasis on established CRISPR-Cas systems and emerging RNA-guided nucleases such as Fanzor. By integrating clinical context with technological progress, we aim to highlight current achievements, ongoing challenges, and future prospects for curative genome-based therapies in SCD.

## 2 Sickle cell disease (SCD)

Sickle cell disease (SCD) is a genetic blood disorder caused by a single base-pair mutation in the beta-globin gene. This mutation involves the substitution of adenine (A) with thymine (T), resulting in the replacement of the hydrophilic amino acid glutamic acid (GAG) with the hydrophobic amino acid valine (GTG), at the sixth position of the beta-globin chain (Figure 1) (Inusa et al., 2019). This mutation leads to HbS polymerization under hypoxic conditions, driving red blood cell deformation, hemolysis, and vaso-occlusive complications (Thalassaemia, 2017).

Clinically, SCD is associated with a wide range of symptoms and complications, including severe pain crisis, chronic anemia, fatigue, jaundice, stroke, and an increased susceptibility to infections, particularly due to splenic dysfunction (Piel et al., 2017; Ware et al., 2017). The blockage of capillaries by sickled cells can lead to life-threatening events and significantly affect patients’ quality of life (Dierick et al., 2025). Importantly, the clinical course of SCD is highly variable among individuals. Some patients experience relatively mild symptoms, while others suffer from frequent, severe complications (Habara and Steinberg, 2016). Despite decades of research, the reasons behind this variability remain largely unclear (Habara and Steinberg, 2016). Attempts to identify reliable genotypic or clinical predictors of disease severity have been inconclusive, and currently, no validated *in vitro* biomarkers exist to guide prognosis or stratify risk (Inusa et al., 2019; Bell et al., 2024).

From a genome-editing perspective, these molecular and clinical features of SCD define several rational targets for CRISPR-based intervention. Direct correction of the pathogenic HBB variant or recreation of benign β-thalassemia or hereditary persistence of fetal hemoglobin (HPFH) alleles at the HBB/HBG loci aims to prevent HbS polymerization at its source (Badat et al., 2023). Alternatively, disruption of BCL11A or its erythroid-specific enhancer, as well as editing of HBG promoters and other fetal hemoglobin regulators, can reactivate γ-globin expression and increase fetal hemoglobin levels, which inhibits HbS polymerization and reduces vaso-occlusion (Psatha et al., 2018; Sharma et al., 2023). Additional candidate targets include genes involved in red cell hydration, adhesion, and inflammation, which could modulate downstream complications (Carden and Little, 2019). Although still in preclinical development, Fanzor-based genome-editing strategies would be expected to pursue the same target classes as CRISPR—HBB correction, HbF reactivation via BCL11A and HBG regulatory regions, and modulation of downstream modifier genes—while potentially offering advantages in delivery and immunogenicity for specific clinical settings (Saito et al., 2023). Together, these strategies illustrate how insights into the molecular pathogenesis of SCD directly inform the selection of CRISPR targets designed to curb or even abolish the clinical manifestations of the disease.

## 3 Diagnostic approaches for sickle cell disease (SCD) in pregnant women and newborns

Sickle cell disease (SCD) is diagnosed across all age groups, including children, adolescents, and adults, using a combination of hematological, biochemical, and molecular techniques. Initial evaluation typically includes complete blood count (CBC) parameters, peripheral blood smear examination, and reticulocyte count, which may indicate hemolytic anemia and characteristic red blood cell morphological abnormalities (Piel et al., 2017). Definitive diagnosis is achieved through hemoglobin analysis methods such as hemoglobin electrophoresis, high-performance liquid chromatography (HPLC), and isoelectric focusing, which enable accurate identification and quantification of hemoglobin variants, including hemoglobin S (HbS) (Arishi et al., 2021). In cases where hemoglobin analysis results are inconclusive or require further clarification, molecular diagnostic methods targeting mutations in the HBB gene are employed (Old, 2003). While these diagnostic approaches are well established, they remain clinically and translationally relevant in the context of genome-editing therapies, as accurate diagnosis and early genetic characterization are essential for patient selection, risk stratification, and therapeutic timing.

Definitive diagnosis through hemoglobin analysis and molecular testing of the HBB gene not only confirms disease status but also provides the genetic resolution required for targeted genome-editing interventions. As CRISPR- and Fanzor-based therapies rely on precise genomic targeting, early identification of affected individuals—particularly during the prenatal and neonatal periods—facilitates long-term therapeutic planning and informed counseling regarding future curative options.

**FIGURE 3:**
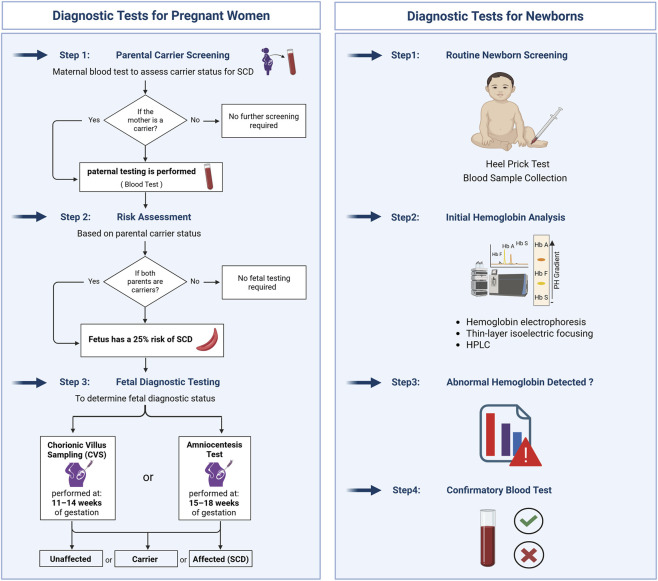
Overview of prenatal and newborn screening pathways for sickle cell disease (SCD), illustrating carrier screening in parents, fetal diagnostic testing during pregnancy, and routine newborn screening for early SCD disease detection.

In pregnancy, carrier screening and fetal diagnostic testing are recommended as early as possible to identify couples at risk and, when indicated, determine whether the fetus is affected (Ward et al., 2018). The initial screening is typically a blood test to determine if the mother is a carrier of SCD or thalassemia (Figure 3). If she is found to be a carrier, the father is also offered testing. If both parents are carriers, there is a 25% chance the fetus will have SCD (Weil et al., 2020; Hussein et al., 2021). If screening results indicate a higher risk, diagnostic procedures may follow. Chorionic villus sampling (CVS), performed between 11 and 14 weeks of gestation, involves collecting a small sample of placental tissue for DNA-based testing of the sickle hemoglobin gene. Alternatively, amniocentesis—typically conducted between 15 and 18 weeks—involves extracting a small amount of amniotic fluid from the uterus to analyze the fetal genetic makeup. These diagnostic procedures can confirm whether the fetus has inherited SCD or another hemoglobin disorder (Figure 3) (Thalassaemia, 2017; Weil et al., 2020; Hussein et al., 2021). These prenatal strategies support early risk assessment, targeted genetic counseling, and informed reproductive decision-making, and they also provide a framework for future *in utero* or very-early-life genome-editing interventions as CRISPR- and Fanzor-based therapies evolve (Abdulraheem Fadhel et al., 2017).

In many countries, newborn screening for SCD is routinely performed using heel-prick blood samples shortly after birth (McGann and Hoppe, 2017). Techniques such as hemoglobin electrophoresis, thin-layer isoelectric focusing, or high-performance liquid chromatography (HPLC) are commonly used to detect abnormal hemoglobin patterns (Arishi et al., 2021). If the initial screening indicates the presence of SCD, a confirmatory blood test is conducted to confirm the diagnosis (Figure 3) (Han and She, 2017). Overall, early identification of SCD supports early disease characterization and long-term therapeutic planning, which is increasingly relevant as curative genome-editing strategies, including CRISPR- and Fanzor-based therapies, progress toward clinical application (Grosse et al., 2011).

## 4 Treatment of sickle cell disease (SCD): context for genome-editing therapies

**FIGURE 4:**
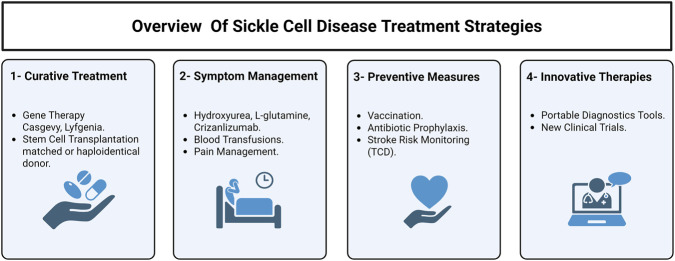
Overview of current and emerging treatment approaches for SCD. Abbreviations: Exacel, Exagamglogene Autotemcel; Lyfgenia, Lovotibeglogene Autotemcel.

The management of SCD has traditionally focused on symptom control, prevention of complications, and reduction of disease-related morbidity and mortality. Conventional therapeutic approaches include pharmacological treatments, blood transfusion programs, and preventive measures aimed at reducing infection risk and vascular complications (Figure 4) (Riley et al., 2024; Ward et al., 2018; Ross et al., 2022; Niihara et al., 2018; Ataga et al., 2017). While such approaches have played an important historical role, they require lifelong treatment and are therefore not curative. These limitations have driven the development of definitive, gene-based therapeutic strategies that directly target the molecular cause of SCD.

Historically, curative treatment options for SCD have been limited to allogeneic hematopoietic stem cell transplantation (HSCT), which replaces the patient’s defective hematopoietic system with donor-derived stem cells. Although effective, HSCT is limited by donor availability, transplant-related toxicity, graft-versus-host disease, and strict eligibility criteria (Bhalla et al., 2023; Kanter et al., 2021; Krishnamurti, 2021). Importantly, HSCT does not inherently involve genome editing, but rather serves as a foundational platform upon which modern gene-editing therapies are built.

Recent advances in *ex vivo* genome editing of autologous hematopoietic stem cells have transformed the therapeutic landscape of SCD. CRISPR-Cas9–based approaches have achieved clinical approval, enabling targeted modification of patient-derived cells to either correct the pathogenic HBB mutation or reactivate fetal hemoglobin expression through disruption of regulatory elements such as BCL11A (Parums, 2024; Leonard and Tisdale, 2024; Ginn et al., 2024). These strategies eliminate the need for matched donors and substantially reduce immunological risks associated with allogeneic transplantation.

Beyond CRISPR-Cas systems, emerging genome-editing platforms such as Fanzor (Fz) offer additional therapeutic potential. Fz’s compact size and eukaryotic origin may provide advantages in delivery efficiency and cellular compatibility, positioning it as a promising next-generation alternative for SCD gene therapy. Collectively, genome-editing technologies represent a paradigm shift in SCD management—from lifelong symptomatic treatment toward definitive, potentially curative interventions—and form the primary focus of the subsequent sections of this review.

### 4.1 Curative treatments

#### 4.1.1 Gene therapy

**FIGURE 5:**
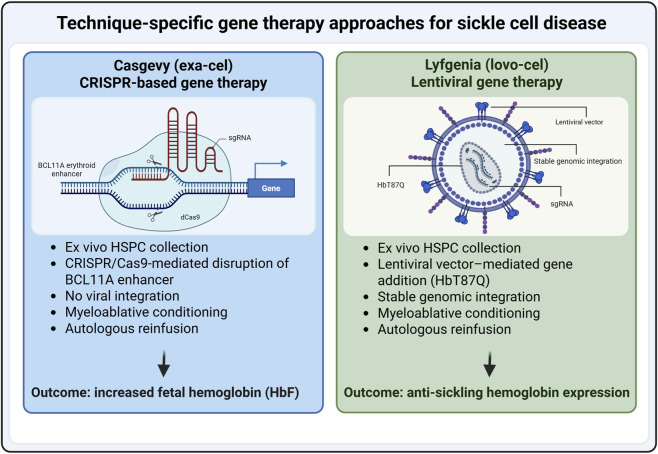
Gene therapy approaches for sickle cell disease. Left panel: CRISPR/Cas9-based genome editing (Casgevy), where autologous CD34^+^ HSPCs are edited at the BCL11A erythroid enhancer to increase fetal hemoglobin, then reinfused after conditioning. Right panel: Lentiviral gene addition (Lyfgenia), where autologous HSPCs are transduced with a vector encoding anti-sickling Hb T87Q and returned to the patient post-conditioning. Both approaches enable durable engraftment of modified HSPCs but differ in whether they precisely edit endogenous sequences or insert an exogenous transgene.

One promising therapy, Casgevy (exagamglogene autotemcel, also known as exa-cel), utilizes CRISPR-based technology (CRISPR/Cas9 gene-editing) to edit a patient’s own stem cells, enabling them to produce healthy, non-sickling red blood cells (Parums, 2024; Leonard and Tisdale, 2024; Casgevy, 2024; England, 2025; CRISPR, 2025a). Casgevy was approved in 2023 for use in the United States (US), United Kingdom, European Union (EU), Saudi Arabia, and several other countries. It is indicated for the treatment of both SCD and transfusion-dependent β-thalassemia in patients aged 12 years and older with recurrent vaso-occlusive crises. Casgevy functions by extracting stem cells from the patient’s bloodstream and genetically modifying them to boost the production of fetal hemoglobin, which helps prevent the sickling of red blood cells (Ginn et al., 2024; Casgevy, 2024). The edited cells are then reinfused into the patient, significantly lowering the risk of immune rejection since they originate from the same individual (Ginn et al., 2024; Casgevy, 2024). Clinical trials have demonstrated remarkable results, with most patients becoming free from painful vaso-occlusive crises, and individuals with β-thalassemia achieving long-term independence from blood transfusions (Ginn et al., 2024; Frangoul et al., 2024). Nonetheless, treatment has been associated with several side effects, including reduced levels of platelets and white blood cells, mouth ulcers, nausea, musculoskeletal and abdominal discomfort, vomiting, febrile neutropenia, headaches, and itching. These outcomes emphasize the need for continued surveillance and further investigation to fully assess the long-term safety and effectiveness of the therapy (Figure 5) (Leonard and Tisdale, 2024; Frangoul et al., 2024).

Another recently approved gene therapy for SCD is Lyfgenia (lovotibeglogene autotemcel, or lovo-cel), which received US Food and Drug Administration (FDA) approval in December 2023 (Commissioner, 2025; Leonard and Tisdale, 2024). In contrast to Casgevy, Lyfgenia uses a lentiviral vector to alter the patient’s own hematopoietic stem cells, enabling them to produce hemoglobin T87Q (Giommetti and Papanikolaou, 2024; Lesmana et al., 2024), a modified form of hemoglobin that resists sickling. Following high-dose chemotherapy for myeloablative conditioning, the engineered cells are reinfused into the patient as a one-time treatment, with many individuals showing signs of a functional cure within 6–18 months (Ginn et al., 2024; Giommetti and Papanikolaou, 2024). The most frequently reported side effects include mouth sores (stomatitis), low counts of platelets, neutrophils, and red blood cells, as well as febrile neutropenia—primarily due to the intensive chemotherapy regimen (Li and Mandal, 2024). Notably, Lyfgenia comes with a US FDA boxed warning because of the potential risk of blood cancers, such as leukemia (Leonard and Tisdale, 2024; Alrehaili et al., 2021). Despite its potential to provide a lasting solution for SCD, Lyfgenia’s serious side effects and long-term safety concerns necessitate careful medical oversight, comprehensive patient education, and continued follow-up to ensure safe and effective use (Figure 5) (Ginn et al., 2024; Giommetti and Papanikolaou, 2024).

Although Casgevy and Lyfgenia represent major breakthroughs in treating SCD, their global accessibility remains severely limited. The burden of SCD is highest in sub-Saharan Africa, the Middle East, and India—areas often constrained by underdeveloped healthcare systems and limited financial resources (Grosse et al., 2011; Elendu et al., 2023; Egesa et al., 2022; Williams, 2016). With treatment costs exceeding USD 2 million per patient, these therapies are largely out of reach for individuals in low- and middle-income countries (LMICs) (Kelkar et al., 2025). Additionally, successful administration requires advanced infrastructure for stem cell collection, *ex vivo* gene editing, myeloablative conditioning, and specialized post-treatment care—services that currently exist only in a few tertiary centers in high-income countries.

### 4.2 Symptom management

#### 4.2.1 Medications

Several pharmacologic agents are used to modify disease course and alleviate symptoms in SCD. Hydroxyurea remains the most established disease-modifying therapy and is often considered the “gold standard,” primarily through induction of fetal hemoglobin, which reduces vaso-occlusive crises, transfusion needs, and other complications (Ward et al., 2018; Ross et al., 2022). Its use requires regular monitoring because of dose-dependent cytopenias and infection risk (Inusa et al., 2019; Ward et al., 2018; Ross et al., 2022; Ingram, 1957). L-glutamine improves redox balance in red blood cells, thereby mitigating oxidative stress and lowering the frequency of pain crises (Niihara et al., 2018), while crizanlizumab, a P-selectin inhibitor, reduces vaso-occlusion by limiting adhesion of sickled cells to the endothelium (Ataga et al., 2017; Karki and Kutlar, 2021; Kanter, 2018; Karki et al., 2022). Collectively, these agents provide important symptomatic and disease-modifying benefits but do not eliminate the underlying genetic defect, underscoring the rationale for curative approaches such as gene therapy and genome editing.

#### 4.2.2 Blood transfusions

Intermittent or chronic transfusion therapy is widely used to correct anemia and reduce the risk of stroke, particularly in children with abnormal transcranial Doppler findings (Guidelines for Red Blood Cell, 1997; Adams et al., 1998). However, long-term transfusion carries risks such as iron overload and alloimmunization, necessitating careful monitoring and chelation therapy when indicated. These limitations further highlight the need for durable, mutation-directed interventions.

#### 4.2.3 Pain management

Pain—especially vaso-occlusive crises (VOC), —is one of the most frequent and debilitating complications of SCD and a leading cause of emergency visits and hospitalization (Cureus, 2025; Tanabe et al., 2007; Simon et al., 2016). Acute VOC-related pain is typically managed with stepwise analgesia (non-opioid agents followed by weak and then strong opioids as needed), whereas chronic pain often requires multimodal strategies that combine pharmacologic treatment with physical and psychosocial interventions (Tanabe et al., 2007; Simon et al., 2016; Iheanyi and Adel, 2002). While these approaches can improve quality of life, they remain largely supportive and do not prevent recurrent tissue injury, reinforcing the need for therapies that address the molecular cause of SCD.

### 4.3 Preventive measures

Preventive strategies aim to reduce infection, stroke, and organ damage and thus remain a cornerstone of comprehensive SCD care. Key components include age-appropriate vaccinations and prophylactic antibiotics to lower the risk of severe bacterial infections such as pneumonia and meningitis (Ahmadi et al., 2015; Mann-Jiles and Morris, 2009). Moreover, early stroke screening using transcranial Doppler ultrasound in children, with regular transfusions for those at high risk (Sickle Cell Anemia, 2025; CDC, 2025). These measures have substantially improved survival and childhood outcomes but require lifelong implementation, further motivating the development of curative approaches that could lessen dependence on intensive supportive care.

### 4.4 Innovations in treatment

Emerging technologies and ongoing clinical trials are revolutionizing SCD care (Patel et al., 2023; Obeagu et al., 2024). Portable diagnostic tools, such as image processing techniques, differentiate healthy red blood cells from sickled cells, offering potential for rapid and cost-effective screening (Arishi et al., 2021). Clinical trials continue to explore new drugs and therapies aimed at improving patient outcomes and advancing treatment strategies (Barak et al., 2024). While symptom management remains the cornerstone of treatment for most SCD patients, advancements in gene therapy and stem cell transplantation offer significant hope for curative solutions (Annals of Blood, 2025). As research continues, improving accessibility to these therapies and expanding treatment options will be crucial to transforming SCD care on a global scale (Barak et al., 2024; Orkin and Bauer, 2019).

## 5 Targeted gene alteration: advances and challenges

Despite advances in symptom management and preventive care for sickle cell disease (SCD), targeted gene alteration has emerged as a strategy to address the disorder at its molecular origin. Historically, targeted gene alteration, such as gene addition, replacement, or deactivation, has been pursued through homologous recombination (HR) (Tan et al., 2016). HR is a highly conserved cellular mechanism that facilitates the exchange of genetic information between DNA molecules by using an intact homologous DNA sequence as a repair template (Tan et al., 2016). This pathway is integral to several critical biological processes, including the repair of double-strand breaks (DSBs) (Figure 2), the rescue of stalled or collapsed replication forks, horizontal gene transfer, and meiosis. These functions are essential for preserving genomic stability and promoting genetic diversity (Kaniecki et al., 2018).

DSBs, which may be either two-ended or one-sided, pose a significant threat to genome stability (Kim and Kim, 2014; Jackson and Bartek, 2009). Failure to repair DSBs or their mis-repair can result in chromosome loss, chromosomal rearrangements, apoptosis, or carcinogenesis (Cho et al., 2014; Kim et al., 2009). Homology-directed repair (HDR), a subtype of HR, is the most accurate mechanism for repairing DSBs, as it relies on a homologous sequence to guide precise repair. When the repair template is identical to the original DNA at the break site, HDR is typically error-free. However, if the template contains specific alterations, HDR can also be harnessed to introduce targeted mutations into the genome (Figure 2) (Li et al., 2020).

With the advent of programmable nucleases such as the CRISPR–Cas system, DSBs can now be deliberately induced at defined loci so that HDR uses an exogenous donor template mimicking the flanking sequence, enabling precise gene correction or insertion of new genetic material in target cells (Figure 2) (Kim and Kim, 2014; Chu et al., 2015). This ability to program DSB location has transformed HDR from a rare, stochastic event into a broadly applicable strategy for precise gene addition and correction in mammalian cells and disease models.

In contrast, the repair process governed by NHEJ is prone to inaccuracies (Figure 2). It often produces insertions or deletions (indels) at the break site, which can result in gene disruption or silencing (Figure 2) (Zhao et al., 2020). Indels occurring within coding regions may lead to frameshift mutations that either promote degradation of the mRNA via nonsense-mediated decay or result in the production of truncated, nonfunctional proteins (Zhao et al., 2020). Despite its lower fidelity, NHEJ is considered less complex than HDR-based strategies for two main reasons: (a) it does not require a donor template, and (b) it operates throughout the entire cell cycle, unlike HDR, which is restricted to the S and G2 phases (Chang et al., 2017). Similarly to RNA interference (RNAi), NHEJ can be applied in stable cell lines to inactivate one or more genes, thereby generating loss-of-function mutations that result in permanent gene silencing (Kim and Kim, 2014).

Recent work has further improved HDR-based editing by using small molecules and cell cycle modulation to increase knock in efficiency several fold and by engineering CRISPR platforms and delivery vehicles that enhance on target activity and reduce genotoxicity. Despite these advances, several challenges remain in targeted gene alteration. Off-target effects, limited HDR efficiency, and delivery barriers continue to restrict precise genome editing. Moreover, the long-term safety and ethical implications of genome manipulation require careful evaluation before broad clinical application.

## 6 Genome-editing technologies: programmable nucleases

**FIGURE 6:**
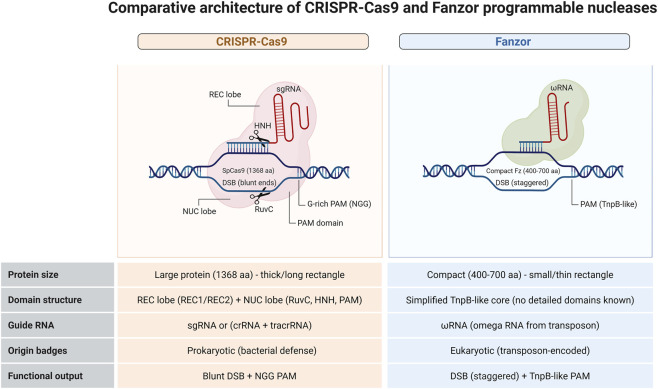
Structural and functional comparison of CRISPR-Cas9 and Fanzor programmable nucleases. CRISPR-Cas9 (SpCas9; 1368 amino acids) comprises multiple functional domains, including REC1 and REC2 regions responsible for sgRNA binding and the RuvC and HNH nuclease domains that mediate blunt-end double-strand DNA breaks at G-rich PAM sites (NGG). In contrast, Fanzor endonucleases (400–700 amino acids) are compact, eukaryotic TnpB-like proteins guided by ωRNA that generate DNA breaks with distinct PAM requirements and cleavage patterns.

The main genome-editing technologies are summarized below, with CRISPR and the recently discovered Fz system illustrated in Figure 6 for comparative purposes.

### 6.1 CRISPR

The CRISPR system is an adaptive prokaryotic immune system that functions as a bacterial defensive mechanism against the insertion of foreign genomic material and the consequences of mobile genetic elements provided by phages and plasmids (Figure 6) (Han and She, 2017; Gholizadeh et al., 2017; Koonin and Makarova, 2019).

The CRISPR/Cas system is divided into two classes: Class I (types I, III and IV) and Class II (types II, V and VI), depending on the structures and functions of the Cas proteins (Parums, 2024; Nishimasu et al., 2014; Bhattacharya and Satpati, 2022; Koonin et al., 2017; Makarova et al., 2020) (Table 1). Class I systems have Cas protein complexes with multiple subunits, while class II systems use a single Cas protein. Among them, type II CRISPR/Cas-9 stands out due to its comparatively simple structure, which has led to extensive studies and use in genetic engineering (CRISPR, 2025b; Asmamaw and Zawdie, 2021; Singh et al., 2025).

**TABLE 1:** Comparative overview of major genome-editing modalities.

Editing tool	Origin	Guide/Recognition	Target type	Cleavage/Edit type	Typical delivery	Key advantages	Main limitations/clinical status
ZFNs (Ma et al., 2023; Rao et al., 2022; Hussen et al., 2024)	Synthetic modular proteins	Engineered zinc-finger domains fused to FokI; protein–DNA recognition	dsDNA	DSB via FokI dimer	Plasmid DNA, mRNA, or protein	First clinically used targeted nucleases; well-defined binding code	Complex protein engineering; off-targets from overlapping motifs; largely superseded by CRISPR/Fz; early trials in CCR5/BCL11A
TALENs (Ma et al., 2023; Rao et al., 2022; Hussen et al., 2024)	Bacterial TALEs (Xanthomonas)	TALE repeats with RVDs fused to FokI; protein–DNA recognition	dsDNA	DSB via FokI dimer	mRNA or protein	High specificity; robust in many cell types; clinically validated in some CAR-T products	Large, repetitive constructs; more complex to engineer than gRNA; limited current use in SCD
CRISPR–Cas9 (Saito et al., 2023; Li et al., 2025; Liu et al., 2025; Bao and Jurka, 2013; Urnov et al., 2010; Silva et al., 2011; Zhang et al., 2014; Frangoul et al., 2021; Saito et al., 2021; Dimitrievska et al., 2024; Lattanzi et al., 2021; Cai et al., 2018; Antoniani et al., 2018; Park and Bao, 2021)	Bacterial adaptive immunity	gRNA (crRNA + tracrRNA or sgRNA) guides Cas9 to PAM-adjacent site	dsDNA	Blunt DSB at target site	Plasmid, mRNA, viral vectors (AAV/lenti), or RNP	Simple retargeting via gRNA; high efficiency; most advanced clinically; FDA-approved for SCD/β-thalassemia	Off-target cuts and genotoxicity risk; large protein (∼1368 aa); mainly *ex vivo* for SCD at present
CRISPR–Cas12/Cas13	Bacterial adaptive immunity	Single crRNA	Cas12: dsDNA; Cas13: ssRNA	Cas12: staggered DSB; Cas13: RNA cleavage	mRNA, protein, or viral vectors	Broader PAM options (Cas12); RNA targeting without permanent DNA change (Cas13); useful in diagnostics	Fewer clinical data than Cas9; collateral activity (Cas13) must be controlled; early translational stage
Base editors	CRISPR-derived	gRNA	dsDNA	No DSB; C→T or A→G base substitution	As for CRISPR (mRNA, viral vectors, RNPs)	Precise point mutations without DSBs; lower indel burden	Limited to certain base conversions; early clinical development
Prime editors	CRISPR-derived	pegRNA (gRNA + RT template)	dsDNA	Nick plus templated repair (insertions, deletions, all base changes)	mRNA, viral vectors, or RNP	Highly versatile precise editing without DSBs; broad mutation scope	Larger constructs; delivery challenges; currently preclinical/early clinical
Fz	Eukaryotic, transposon-encoded (TnpB-like)	ωRNA-guided recognition	dsDNA	RNA-guided DSB or nick (under study)	DNA, mRNA, or protein; amenable to viral or nanoparticle delivery	Compact (400–700 aa); eukaryotic origin may improve expression and immunogenicity profile; expands RNA-guided toolbox beyond Cas	Editing rules and specificity still being defined; efficiency under optimization; preclinical only

Abbreviations: AAV, adeno-associated virus; DSB, double-strand break; dsDNA, double-stranded DNA; fz, Fanzor; gRNA, guide RNA; HSPC, hematopoietic stem and progenitor cell; lenti, lentivirus; PAM, protospacer adjacent motif; pegRNA, prime editing guide RNA; RNP, ribonucleoprotein; RVD, repeat-variable di-residue; SCD, sickle cell disease; sgRNA, single guide RNA; ssRNA, single-stranded RNA; ωRNA, omega RNA; ZFNs, zinc finger nucleases; TALENs, transcription activator-like effector nucleases.

The CRISPR/Cas-9 system consists of two key components: the guide RNA (gRNA) and the CRISPR-associated (Cas-9) proteins (Asmamaw and Zawdie, 2021). The main Cas protein used in genome editing, Cas-9, is originally derived from *Streptococcus pyogenes* (SpCas-9). It consists of 1368 amino acids and functions as a large multidomain DNA endonuclease, often referred to as “genetic scissors”, by cleaving the target DNA and creating a double-strand break (CRISPR, 2025b; Gao et al., 2019; Le Rhun et al., 2019; Varble et al., 2022) (Table 1).

Cas-9 has two main regions: the recognition lobe (REC) and the nuclease lobe (NUC). The REC lobe comprises the REC1 and REC2 domains, which bind to the guide RNA (Nishimasu et al., 2014; Bhattacharya and Satpati, 2022). In contrast, the NUC lobe consists of the interaction domains RuvC, HNH and Protospacer Adjacent Motif (PAM). The HNH and RuvC are nuclease that cleave the target sequence, while the PAM interaction domain provides PAM specificity and initiates binding to the target DNA (Table 1) (Li et al., 2025; Liu et al., 2025).

The guide RNA consists of two components: CRISPR RNA (crRNA) and trans-activating CRISPR RNA (tracrRNA) (Asmamaw and Zawdie, 2021). The crRNA, generally 18–20 base pairs long, identifies the target DNA by binding to the complementary sequence (Hsu et al., 2014; Jinek et al., 2012). The tracrRNA, on the other hand, is a long sequence of loops that serves as a binding scaffold for the Cas-9 nuclease (Nishimasu and Nureki, 2017). In prokaryotes, guide RNA targets viral DNA, but in gene editing applications, crRNA and tracrRNA can be synthesized together to create a single guide RNA (sgRNA) that can be used to edit virtually any gene sequence (Hsu et al., 2014; CRISPR, 2025b; Jinek et al., 2012; Science, 2017) (Table 1).

Beyond Cas9, other Cas nucleases have been characterized with unique features and expanded editing capabilities. Cas12 (also known as Cpf1) belongs to the Class II, Type V CRISPR system and targets double-stranded DNA through a distinct mechanism (Table 1). Unlike Cas9, which produces blunt-ended cuts, Cas12 generates staggered (“sticky”) ends by cleaving the non-target strand several nucleotides upstream of a T-rich PAM sequence (e.g., TTTV), broadening its target range beyond the G-rich PAM requirement of Cas9. Moreover, Cas12 utilizes a single crRNA instead of the dual crRNA–tracrRNA complex, simplifying its design and delivery for genome editing. Cas12 variants, such as Cas12a and Cas12f, have demonstrated efficient and precise genome modification in both prokaryotic and eukaryotic systems (Paul and Montoya, 2020). In contrast, Cas13, belonging to the Class II, Type VI CRISPR system, uniquely targets single-stranded RNA rather than DNA, enabling transient and reversible regulation of gene expression in eukaryotic cells. Guided by a crRNA, Cas13 exhibits RNase activity that cleaves both target and collateral RNAs, a property that has been leveraged in RNA diagnostic platforms such as SHERLOCK and REPAIR. Its RNA-targeting ability allows for modulation of gene expression and detection of RNA viruses without permanent genomic alterations (Xu and Li, 2020) (Table 1).

In addition to Cas nucleases, several emerging genome-editing approaches have been developed, including base editing, prime editing, integrase-mediated editing, and bridge recombinase technologies. Base editing allows for precise, programmable conversion of individual nucleotides without generating double-strand breaks, minimizing unintended mutations (Cabré-Romans and Cuella-Martin, 2025). Prime editing further expands this precision by combining a Cas9 nickase with reverse transcriptase, enabling targeted insertions, deletions, and all twelve possible point mutations with reduced off-target effects (Li et al., 2025). Integrase-mediated and bridge recombinase methods offer alternative, sequence-specific DNA integration and recombination strategies that do not rely on nucleases, potentially improving safety and efficiency. Although still in early stages and less clinically advanced than CRISPR-Cas systems, these techniques broaden the genome-editing toolkit and hold promise for treating a wider array of genetic diseases with greater specificity and minimal genomic disruption (Liu et al., 2025).

### 6.2 Fanzor (Fz)

Fanzor (Fz) is a eukaryotic TnpB-IS200/IS605-like protein that is encoded by transposable elements (Saito et al., 2023). It was initially proposed that Fz proteins, along with prokaryotic TnpBs, play a role in regulating the activity of transposable elements, potentially through methyltransferase functions. More recently, TnpB has been recognized as a component of a novel class of RNA-guided systems known as OMEGA (Saito et al., 2023; Bao and Jurka, 2013). OMEGA systems consist of an RNA-guided endonuclease protein, such as TnpB, IscB, or IsrB, in conjunction with a non-coding RNA (ncRNA) that is transcribed from the terminal region of the transposon, referred to as ωRNA. These OMEGA systems are considered to be precursors to CRISPR–Cas systems, with TnpB evolving into the singular RNA-guided endonuclease Cas12 (Altae-Tran et al., 2021; Saito et al., 2023). Additionally, TnpB exhibits distant homology with Fz (Saito et al., 2023). These discoveries suggest that Fz may represent a eukaryotic variant of the CRISPR–Cas or OMEGA systems.

Fz differs from existing CRISPR–Cas systems and other genome-editing approaches in several key ways (Table 1). Unlike Cas9, Cas12, or Cas13, which are derived from prokaryotic adaptive immune systems, Fz is naturally encoded in eukaryotic genomes, potentially improving cellular compatibility, expression, and nuclear localization. Its compact size makes delivery via viral vectors or nanoparticles more feasible than larger Cas proteins (Saito et al., 2023) (Table 1). Mechanistically, Fz uses ωRNA to guide DNA cleavage, similar to CRISPR nucleases, but may offer distinct targeting rules and cleavage patterns (blunt vs. staggered cuts) that are still being characterized (Saito et al., 2023). Compared to protein-only nucleases like ZFNs, TALENs, or meganucleases, Fz’s RNA-guided programmability simplifies engineering while potentially reducing off-target effects (Table 1).

Overall, Fz complements existing genome-editing technologies by providing a lightweight, eukaryote-optimized, RNA-guided platform that broadens the RNA-guided nuclease repertoire beyond prokaryotic Cas enzymes, offering new opportunities for precise and versatile genome manipulation. For Fz to advance toward clinical application, a clear roadmap of experimental validation, preclinical studies, and regulatory milestones is essential, with improvements in editing efficiency and target specificity in relevant cell types being critical priorities for this emerging technology.

### 6.3 Other legacy genome-editing platforms

Earlier genome-editing technologies, including zinc finger nucleases (ZFNs), transcription activator-like effector nucleases (TALENs), and meganucleases, have contributed to foundational advances in gene-editing research and provided important proof-of-concept for targeted genome modification (Urnov et al., 2010; Silva et al., 2011; Zhang et al., 2014). However, their clinical application in sickle cell disease has been limited by challenges related to design complexity, delivery efficiency, scalability, and off-target effects. With the advent of CRISPR-based systems, which offer greater programmability and editing efficiency, and the recent emergence of Fanzor (Fz) as a compact eukaryotic genome-editing platform, these earlier technologies have largely been superseded in the context of therapeutic development (Frangoul et al., 2021; Saito et al., 2021). As such, ZFNs, TALENs, and meganucleases are now primarily of historical and comparative interest, while current research and clinical translation efforts are predominantly focused on CRISPR-and Fz-based approaches (Table 1).

### 6.4 Limitations of CRISPR in SCD treatment

Despite the promising clinical outcomes achieved with Genome-Editing therapies for sickle cell disease, several limitations remain. One of the primary concerns is the risk of off-target genome editing, which may lead to unintended genetic alterations with potential long-term consequences. Although advances in guide RNA design and high-fidelity Cas variants have reduced this risk, complete elimination of off-target activity remains challenging (Kosicki et al., 2018; Tsai and Joung, 2016). Efficient delivery of CRISPR components to hematopoietic stem and progenitor cells also presents a significant hurdle. Current approaches rely largely on *ex vivo* manipulation followed by autologous stem cell transplantation, a process that is technically complex, resource-intensive, and not universally accessible. In addition, variability in editing efficiency and stem cell engraftment may influence therapeutic durability and clinical outcomes (Table 1) (Frangoul et al., 2021; Dever et al., 2016). Furthermore, long-term safety and durability of gene editing remain areas of active investigation. Persistent expression of edited cells, potential genotoxicity, and unknown late adverse effects necessitate prolonged clinical follow-up. Furthermore, the high cost, specialized infrastructure requirements, and regulatory complexity associated with CRISPR-based therapies limit their global scalability, particularly in low-resource settings where SCD burden is highest (Table 1) (Romero et al., 2019; Cornu et al., 2017). These challenges underscore the need for continued innovation in genome-editing technologies and support the exploration of alternative platforms, such as Fz-based systems, which may offer advantages in delivery efficiency and cellular compatibility.

## 7 Clinical approvals and translational applications of CRISPR and fanzor in sickle cell disease

### 7.1 CRISPR clinical applications in SCD

CRISPR-Cas9 gene editing has emerged as a transformative tool in the treatment of SCD, offering two main strategies: gene correction and gene disruption (Dimitrievska et al., 2024). Gene correction focuses on repairing the underlying mutation in the HBB gene responsible for SCD (Lattanzi et al., 2021; Cai et al., 2018). Using CRISPR-Cas9, precise edits are introduced to replace the defective sequence with a healthy version, thereby restoring normal hemoglobin (HbA) production (Dimitrievska et al., 2024; Antoniani et al., 2018; Park and Bao, 2021; Desai et al., 2024). One notable example is the CRISPR_SCD001 therapy, which utilizes electroporation to deliver the CRISPR components directly into hematopoietic stem cells (HSPCs) without the need for viral vectors (Ugalde et al., 2023; Ma et al., 2023). These edited stem cells are then transplanted back into the patient, enabling the sustained production of healthy red blood cells and offering the potential for a lifelong cure (Dimitrievska et al., 2024; Rao et al., 2022; Hussen et al., 2024). Similar approaches using either HSCs or induced pluripotent stem cells (iPSCs) have shown promise in restoring HbA levels and reducing sickle hemoglobin (Lattanzi et al., 2021; Park and Bao, 2021; Ou et al., 2016).

On the other hand, gene disruption aims to reactivate fetal hemoglobin (HbF) production, which can inhibit sickling of red blood cells (Dimitrievska et al., 2024; Fathallah and Atweh, 2006; Steinberg et al., 2014; Quinn and Ware, 2025). This is achieved by targeting regulatory elements like the BCL11A enhancer or γ-globin promoters, disrupting their function to increasing HbF expression (Traxler et al., 2016; Weber et al., 2020). For instance, editing the LRF binding sites or the promoter regions of the HBG1 and HBG2 genes has been shown to enhance HbF synthesis in patient-derived HSPCs, significantly reducing sickling and alleviating symptoms (Dimitrievska et al., 2024; Wienert et al., 2015). Targeting genes like BCL11A—a key repressor of γ-globin—has proven especially effective, with studies demonstrating increased γ-globin levels and improved SCD phenotypes (Frangoul et al., 2021; Dimitrievska et al., 2024; Khosravi et al., 2019; Canver et al., 2015). Additionally, removing segments containing the δ- and β-globin genes can further reactivate HbF synthesis (Antoniani et al., 2018; Khosravi et al., 2019). While several targets have been explored, disrupting major regulators like BCL11A and KLF1 appears to yield the most therapeutically relevant increases in HbF (Lesmana et al., 2024; Lamsfus-Calle et al., 2020; Gillinder et al., 2020).

Both gene correction and gene disruption approaches have shown encouraging results in preclinical models and early clinical trials, fueling optimism for their long-term impact on SCD management (Frangoul et al., 2021; Dimitrievska et al., 2024; Ribeil et al., 2017). The translation of these advances into clinical practice reached a major milestone with the recent approval of Casgevy by the Saudi Food and Drug Authority (SFDA) (Singh et al., 2017; Casgevy, 2025). Developed by Vertex Pharmaceuticals in collaboration with CRISPR Therapeutics, Casgevy is a groundbreaking therapy that edits the disease-causing gene in patient-derived stem cells. The process involves extracting bone marrow stem cells, editing them in the laboratory to correct the genetic defect, and re-infusing them for sustained therapeutic benefit (Singh et al., 2017; Casgevy, 2025).

Despite these remarkable advances, several challenges remain. Concerns about off-target effects, long-term safety, and the optimization of delivery methods continue to be the focus of ongoing research (Rees et al., 2010; Ingram, 1957). Active clinical trials are essential to further evaluate the efficacy and safety of CRISPR-Cas9-based therapies (Singh et al., 2017; Zarghamian et al., 2022), ultimately shaping their future role in SCD treatment and determining their potential to provide a definitive cure or sustained therapeutic benefit.

### 7.2 Fanzor translational potential in SCD

Fanzor (Fz), a recently discovered RNA-guided DNA-cutting enzyme found in eukaryotes, holds significant promise for treating genetic disorders, including SCD (Saito et al., 2023; Wu et al., 2024). This innovative gene-editing technology offers distinct advantages over existing CRISPR-Cas systems, positioning it as a potential game-changer in therapeutic applications. Fz lies in its ability to expand the genome-editing toolbox beyond CRISPR by providing a compact, programmable nuclease that could be better suited for certain *in vivo* applications rather than acting as a direct replacement for Cas9.

One key advantage is its compact size, ranging from 400 to 700 amino acids compared to the much larger CRISPR-Cas systems (1000–1600 amino acids) (Saito et al., 2023). This smaller structure greatly enhances vector compatibility—particularly with adeno-associated virus serotype 6 (AAV6), a vector commonly used for efficient transduction of hematopoietic stem and progenitor cells (HSPCs) (Hirsch et al., 2016; Wang et al., 2019). The reduced coding sequence of Fz allows more efficient packaging within the limited cargo capacity of AAV6 (∼4.7 kb), facilitating higher delivery efficiency, lower vector load, and improved gene-transfer fidelity in HSPCs compared to bulkier CRISPR-Cas9 constructs (Hirsch et al., 2016; Wang et al., 2019). From a translational perspective, this size advantage positions Fz as a promising candidate for next-generation *in vivo* genome-editing strategies, which remain a major unmet need in current SCD gene therapy.

Additionally, certain Fz proteins, particularly those derived from fungi, exhibit minimal “collateral activity,” reducing the risk of accidental degradation of nearby RNA or DNA and enabling more precise and targeted gene editing (Saito et al., 2023). Like CRISPR, Fz is highly programmable and can be tailored to target specific genomic sites, making it versatile for addressing a variety of genetic disorders (Saito et al., 2023). Importantly, Fz’s eukaryotic origin represents a conceptual shift in therapeutic genome editing, as it may reduce immunogenicity and biosafety concerns associated with bacterial-derived nucleases, potentially facilitating regulatory approval and broader public acceptance.

Although Fz has not yet been specifically tested for SCD, its capabilities suggest potential applications similar to current gene-editing strategies (Saito et al., 2023). These include silencing genes such as BCL11A to boost fetal hemoglobin production, a strategy known to alleviate SCD symptoms; directly correcting the SCD-causing mutation in the HBB gene; and engineering a patient’s hematopoietic stem cells (HSPCs) for autologous cell therapy, which avoids donor dependence and reduces the risk of rejection (Abraham and Tisdale, 2021; Newby et al., 2021). Taken together, these features indicate that Fz could, in principle, support more compact and potentially less immunogenic editing platforms for hemoglobinopathies, especially if its activity in human HSPCs can be brought closer to that of clinically validated CRISPR-Cas9 systems.

Despite these attractive features, several hurdles must be overcome before Fz can be translated into routine clinical practice (Saito et al., 2023). Current data suggest that its editing efficiency in human cells remains inferior to that of established CRISPR nucleases, although protein engineering has already achieved order-of-magnitude improvements in activity (Saito et al., 2023). Rigorous characterization of on-target performance, off-target profiles, and long-term genomic safety will be required to determine whether Fz can meet regulatory standards for first-in-human studies. In addition, practical delivery solutions for tissue- or cell type–specific targeting remain to be fully developed, particularly for *in vivo* HSPC editing (Saito et al., 2023).

From a regulatory and ethical standpoint, the eukaryotic origin of Fz may ultimately prove advantageous, but these benefits remain speculative until supported by systematic immunogenicity and safety data in relevant preclinical models and, eventually, clinical trials. Moreover, as with other genome-editing technologies, responsible implementation of Fz will require careful governance around germline risk, robust containment of off-target events, and deliberate attention to global equity, especially for populations disproportionately affected by SCD.

In this context, Fz is best viewed not as a near-term replacement for CRISPR–Cas9, but as a complementary platform whose strengths may become increasingly relevant as the field moves toward scalable, *in vivo*, and potentially less immunogenic editing solutions. Strategically, its delivery flexibility and compact size could help bridge the gap between current *ex vivo* HSPC editing paradigms and future *in vivo* approaches that aim to improve accessibility and reduce cost. Ultimately, Fanzor’s therapeutic impact will depend on whether it can translate its biophysical and immunological advantages into robust, safe, and ethically governed interventions that broaden the reach of gene therapy beyond what is currently achievable with CRISPR alone.

## 8 Conclusion

SCD remains a major global health problem, with high morbidity, premature mortality, and limited access to curative options such as matched-donor transplantation in many high-burden regions (Kato et al., 2018). At its core, SCD is a single–nucleotide disorder of the HBB gene, yet it drives complex downstream pathobiology involving hemoglobin polymerization, vaso-occlusion, hemolysis, and progressive multi-organ damage, highlighting the need for therapies that directly target the molecular defect while also being scalable and accessible (Bell et al., 2024; Kato et al., 2018).

Against this backdrop, advances in genome editing—most notably CRISPR–Cas9—have fundamentally changed what is possible for monogenic diseases by enabling precise correction of the HBB mutation or durable upregulation of fetal hemoglobin in autologous hematopoietic stem and progenitor cells. Early clinical successes of *ex vivo* CRISPR-based products demonstrate that long-term disease modification and even functional cure are achievable, but they also expose important scientific and translational challenges, including off-target activity, insertion–deletion–mediated genotoxicity, conditioning-related toxicity, and the logistical and economic barriers inherent to complex cell-therapy workflows (Cho et al., 2014). These limitations underscore the need for continued engineering of CRISPR systems, development of safer conditioning and delivery strategies, and parallel exploration of alternative platforms.

In this context, Fz emerges as a promising, though still early-stage, RNA-guided nuclease of eukaryotic origin that could complement rather than replace CRISPR (Saito et al., 2023). Its compact size and potential for lower immunogenicity may offer advantages for vector design, *in vivo* delivery, and regulatory acceptance, particularly if its editing efficiency and specificity in human HSPCs can be brought closer to that of clinically validated CRISPR editors. At present, however, Fz remains preclinical, and its true value will depend on rigorous head-to-head evaluation of on-target potency, off-target profiles, manufacturability, and safety relative to established CRISPR-based approaches.

Looking ahead, the most realistic path toward durable and widely accessible cures for SCD is likely to involve a diversified toolkit in which mature CRISPR-Cas9 therapies and next-generation systems such as Fz are matched to specific clinical, logistical, and regulatory contexts. Key scientific priorities include refining editing tools to minimize genotoxicity, enabling efficient *in vivo* or minimally intensive *ex vivo* HSPC editing, and developing robust long-term safety surveillance. Equally important are translational and access-related challenges: reducing cost and infrastructure requirements, adapting platforms for use in low- and middle-income countries where SCD is most prevalent, and embedding strong ethical and regulatory frameworks that address germline risk and equitable access. If these scientific and implementation hurdles can be overcome, genome editing—anchored by CRISPR and potentially extended by Fz—has the potential not only to transform individual outcomes but also to narrow long-standing global disparities in the care of SCD and related genetic disorders.

## 9 Simple summary

Genome editing has revolutionized the treatment landscape for genetic disorders such as sickle cell disease (SCD), a severe inherited blood disorder that causes chronic pain, organ damage, and shortened lifespan. The recent approval of CRISPR-Cas9-based therapies for severe SCD represents a major advancement in genetic medicine. However, CRISPR technology still faces several technical limitations that call for further innovation. Emerging alternatives, such as the Fanzor (Fz) system, may address some of these challenges. Unlike CRISPR, which originates from prokaryotes, Fz is encoded in the eukaryotic genome, providing a universal RNA-guided mechanism with potentially improved delivery and editing efficiency in human cells. In this review, we discuss the progress and limitations of CRISPR technology in correcting mutations associated with SCD and explore the potential of the Fz system as a novel genome-editing tool for future therapeutic applications.
